# The Optimal Ambient Conditions for World Record and World Class Performances at the Berlin Marathon

**DOI:** 10.3389/fphys.2021.654860

**Published:** 2021-05-28

**Authors:** Volker Scheer, David Valero, Elias Villiger, José Ramón Alvero Cruz, Thomas Rosemann, Beat Knechtle

**Affiliations:** ^1^Ultra Sports Science Foundation, Pierre-Bénite, France; ^2^Institute of Primary Care, University Hospital Zürich, Zürich, Switzerland; ^3^Departamento de Fisiología Humana, Histología, Anatomía, Patológica y Educación Física y Deportiva, Universidad de Málaga, Málaga, Spain; ^4^Medbase St. Gallen Am Vadianplatz, St. Gallen, Switzerland

**Keywords:** running, heat, cold, rain, performance, environmental conditions

## Abstract

The “Berlin Marathon” is the fastest marathon racecourse in the world and has witnessed 11 world records (WRs; eight in men and three in women). Weather conditions can have an important impact on race time and we therefore examined the influence of environmental conditions (i.e., temperature, sunshine, precipitation, barometric pressure, and cloud cover) on WRs and elite (i.e., winner, top three and top 10 finishers) marathon performances of men and women at the “Berlin Marathon” between 1974 and 2019. Average world record marathon times in men were 2:03:52 ± 0:01:19 h:min:s and 2:25:05 ± 0:08:25 h:min:s in females (*p* < 0.05). Male competitions were held 44 times (mean winning time: 2:09:48 ± 0:09:15 h:min:s) and female competitions 41 times (mean winning time: 02:30:35 ± 0:19:09 h:min:s; *p* < 0.05). World record performances were set at mean temperatures of 18.61 ± 2.59°C for men and 13.07 ± 4.01°C for women (*p* > 0.05). The ideal environmental conditions for world record performances for men were temperatures of 18.61°C (*p* > 0.05), sunny, mostly dry days, with higher atmospheric pressure and little cloud cover (all *p* > 0.05). In women, ideal conditions for world records performances were temperatures of 13.07°C (*p* > 0.05), with low atmospheric pressure (*p* > 0.05), but significantly more rain (*p* < 0.05), and with no sunshine (*p* < 0.05) and cloud cover (*p* < 0.05). With elite performances, the ideal temperatures were of 17.36 ± 4.33°C for men and 17.93 ± 4.07°C for women (*p* > 0.05), with little to no rain, and moderate cloud cover and sunshine (*p* > 0.05). In summary, novel findings are, that environmental conditions in world records performances differ between men and women, with women obtaining world records in bad weather (with rain, cloud cover, and no sunshine) and men in good weather (sunny, mostly dry days, with little cloud cover). Larger sample sizes are needed to examine sex differences and environmental conditions on world record marathon performances.

## Introduction

Marathon running (distance of 42.195 km) is the longest endurance running discipline in athletics and the Olympics. Historically, the origins of marathon running often refers to the Greek runner Pheidippides, who returned from Marathon to Athens to deliver the victory message over the Persians in the battle of Marathon; however, this is largely thought to be a fictional story, based on a poem from Robert Browning in 1879. The true historical origin probably dates back to the text of Herodotus, where Pheidippides ran from Athens to Sparta and back during the battle of Marathon (490 BC; Grogan, 1981; Scheer, 2019). Since its inaugural competition during the Olympic Games in Athens in 1896, marathon running has seen a stark increase in participation, not only in elite but also in recreational runners (Knechtle et al., 2018).

Performance aspects are well researched in marathon running, especially the physiological determinants, genetics, training, nutritional, technology and logistical aspects (Joyner, 1991; Larsen, 2003; Jones et al., 2020; Joyner et al., 2020), as well as the importance of optimal pacing strategies (Angus, 2014, 2019; Nikolaidis and Knechtle, 2018). However, external determinants, such as environmental conditions, like ambient temperature, wind, cloud cover, barometric pressure, and precipitations are also important and can have an important impact on marathon performance (Montain et al., 2007; Ely et al., 2008; Knechtle et al., 2019). Indeed, it has been shown among the World Marathon Major races (i.e., Boston, London, Berlin, Chicago, and New York), that weather conditions have important effects on race times and they may be more important than race profile (Maffetone et al., 2017).

The weather variable with the strongest influence on marathon race performance is ambient temperature (Suping et al., 1992; Ely et al., 2007a,b). In elite marathon runners, the ideal temperatures seem to be around 10–12°C, both in men and women (Martin and Buoncristiani, 1999; Ely et al., 2007b); however, even temperatures as low as around 8°C can lead to fast marathon times (Trapasso and Cooper, 1989). Warm weather conditions seem to slow down marathon performances, both in men and women; however, slow runners seem to be more negatively impacted than elite runners (Ely et al., 2007b).

Female athletes may be performing better in cooler ambient temperatures than men, but there is no definite evidence for this (Martin and Buoncristiani, 1999; Ely et al., 2007b). It has been shown that thermal comfort and rating of perceived exertion were significantly higher during exercise in males than in females with ice slurry ingestion (Iwata et al., 2020).

The presence of cloud cover or low solar load is often cited as being ideal conditions for running fast marathons; however, there is no conclusive evidence and it may be that in elite marathon runners this does not even impact on performance times (Bodishbaugh, 1988; Ely et al., 2007b). However, cloud cover and/or solar radiation can be additional factors contributing to the likelihood of running a fast marathon race (Hughson, 1980; Morgan, 1980; Ely et al., 2007b). Wind speed can also have an impact on performance, especially tail wind can improve race performance (Knechtle et al., 2019), whereas headwind or strong tail wind can impair performance (Miller-Rushing et al., 2012; Knechtle et al., 2019), although this seems to depend on the marathon race course, as wind speed had no influence on race performance at the Stockholm marathon (Vihma, 2010).

The marathon with the largest numbers of world records (WRs) and world class performances is the “Berlin Marathon” (Robinson, 2019). The “Berlin Marathon” was founded in 1974 and there have been slight changes to the race course. Events prior to 1990 were limited to West Berlin. Since the German reunification, the race course covers the whole city of Berlin. However, the course profile remained then largely unchanged. There have been 11 world record performances recorded, as well as the first sub 2:05 h:min, sub 2:04 h:min, and sub 2:03 h:min marathon performances (Robinson, 2019). Eilud Kipchoge from Kenya is the current world record marathon holder with a time of 2:01:39 h:min:s during the 2018 edition of the “Berlin Marathon” (Robinson, 2019). In 2020, the “Berlin Marathon” was canceled for the first time since inception, due to the COVID-19 pandemic (Scheer et al., 2021). The “Berlin Marathon” is unique and therefore ideally suited to investigate the potential influence of ambient conditions on world records and elite athletes’ performance. Indeed, it is preferable to investigate environmental conditions at one marathon location, so that data cannot be influenced by other, non-weather factors such as marathon racecourse. To date, no study ever investigated the influence of environmental conditions on world records and elite performance times at the “Berlin Marathon.”

Therefore, the aim of the present study was to investigate the influence of environmental conditions such as temperature, sunshine duration, precipitation, barometric pressure, and cloud cover on world records and elite marathon race performances (i.e., winner, top three and top 10 finishers) at the “Berlin Marathon” (1974 until 2019). In addition, we sought to describe the ideal weather conditions for world record performances and compare performances between sexes. We hypothesized that temperatures of ~10–12°C would be the optimum temperature for both women and men to achieve a world record and elite performance in the “Berlin Marathon” and that fast marathon times were achieved in sunny weather, with little cloud cover and precipitation.

## Materials and Methods

### Ethical Approval

This study was approved by the Institutional Review Board of Kanton St. Gallen, Switzerland, with a waiver of the requirement for informed consent of the participants as the study involved the analysis of publicly available data.

### The Race

The first marathon in Berlin was held 1974 as “1. Berliner Volksmarathon.” At that time, the race was started before the “Mommsenstadion” and the course led to “Grunewald” until the “Strandbad Wannsee” with the finish again before the “Mommsenstadion.” The race took place in the forest of “Grunewald” and passed the lake of “Wannsee.” In contrast to the actual city marathon “BMW Berlin Marathon,” the course was at that time a commute in the “Grunewald.” In 1975, the course was changed with both start and finish in the “Mommsenstadion.” In 1981, the course moved to the city of Berlin with the start before the “Reichstagsgebäude,” leading to “Checkpoint Charlie” and finishing on the “Kurfürstendamm.” In 1987, the start was moved to “Strasse des 17. Juni.” In 1990, the course crossed for the first time the “Brandenburger Tor.”^1^ The race start has changed in the history of “Berlin Marathon.”

In 1977, when the first female world record was set in Berlin, the “4. Berlin Marathon” was started in the morning at 09:00 a.m., whereas the German Championship was started in the afternoon (02:45 p.m. for men and 04:15 p.m. for women). The race course was a “Double Shuttle Race” and was held nearby and within the forest.^2^

### Data Set and Data Preparation

Data (i.e., year of performance, finishing times, and place) were obtained directly from the website of the “Berlin Marathon”^3^ in JSON format and then convert into an Excel file using a custom Python script.

Weather data on race day was obtained from the website of “Deutscher Wetterdienst” by using https://opendata.dwd.de/climate_environment/CDC/observations_germany/climate/hourly/ and then to air temperature, sunshine, precipitation, barometric pressure, and cloud cover.

For historic data, the appropriate location has to be selected by using www.dwd.de/DE/leistungen/klimadatendeutschland/statliste/statlex_html.html?view=nasPublication&nn=16102. We selected weather station 433, which is located at the old West-Berlin airport Tempelhof for data for all years since this weather station covered all years of the race. The maximal distance of this weather station to the start/finish area and/or the race course was between 8.3 and 14 km. We selected the time of the measurement respecting the start time of the marathon (i.e., air temperature 2 h after the start) since the world record in 1977 was set in the afternoon. Sunshine (duration in hours), precipitation (mm), cloud cover (duration in hours), and atmospheric pressure (mbar) were selected for the whole race day. Weather type was classified according to Woś by thermal temperature types as warm, when mean temperatures were between 10.1 and 15°C, cloudiness (sunny, cloudy, and very cloudy) and precipitation (Piotrowicz and Ciaranek, 2020).

### Data Processing

Two data files were used in this study: first, a register of the finishing times in the format h:min:s, along with their sex, and the year of the marathon, and second, a register of the weather data. These files were visually inspected in an Excel spreadsheet first, and then uploaded into a Google Colab notebook, where Python was used to perform the statistical processing and to create the results.

### Statistical Analysis

Descriptive statistical analyses were performed on four nested performance tiers: world record, elite (subgroups: winner, top three, and top 10 finishers) for males and females, separately, both on race finish times and weather variables. The resulting values are presented in terms of their average value (mean) and SD and maximum and minimum values for each category, For the finish times results, also the 25, 50, and 75%, quartiles are included. The finish time normal distributions for the full sample (male and female) are proven graphically, however, the downsampling into world records, elite, top three and top 10 results in non-normal samples, as it similarly occurs with the weather factors. We have therefore used the Kolmogorov-Smirnov two-sample test, which is independent of the distributions and sizes of the samples, whenever our results required of statistical significance analysis. Statistical significance was set at 5% (*p* < 0.05). All analyses were carried out using the Python programming language (Python Software Foundation, www.python.org/), and a Google Colab notebook.^4^

## Results

World record marathon performances were set eight times for male and three times for female athletes at the “Berlin Marathon” and finishing times with their corresponding weather data are shown in Table 1. Male competitions were held 44 times and female competitions 41 times during the observational period and the corresponding marathon finishing times by performance groups and gender, expressed as mean, SD, 25, 50, 75% quartile, and minimum and maximum in h:min:s are shown in Table 2.

**Table 1:** Marathon world record performances by year, gender, and weather conditions.

Year	Gender	Nationality	Time (h:min:s)	Temperature (°C)	Sunshine (h)	Cloud cover (h)	Precipitation (mm)	Atmospheric pressure (mbar)
1998	Male	BRA	2:06:05	19.8	9.9	1.0	0.0	1026.8
2003	Male	KEN	2:04:55	17.6	7.4	4.3	9.8	1005.9
2007	Male	ETH	2:04:26	15.9	0.8	7.0	0.0	1012.5
2008	Male	ETH	2:03:59	18.4	10.6	2.2	0.0	1013.6
2011	Male	KEN	2:03:38	20.4	11.3	0.8	0.0	1010.3
2013	Male	KEN	2:03:23	14.2	10.7	1.4	0.0	1008.9
2014	Male	KEN	2:02:57	21.2	9.8	4.5	0.0	1016.4
2018	Male	KEN	2:01:39	21.4	7.8	5.6	0.0	1013.8
1977	Female	FRG	2:34:48	8.9	0.0	8.0	11.2	1013.1
1999	Female	KEN	2:20:43	16.9	0.0	6.3	1.2	997.7
2001	Female	JPN	2:19:46	13.4	0.0	7.5	4.5	1006.0

BRA, Brazil; KEN, Kenya; ETH, Ethiopia; FRG, Federal Republic of Germany; and JPN, Japan.

**Table 2:** Marathon finishing times by performance groups and gender, expressed as mean, SD, 25, 50, 75% quartile and minimums and maximum in h:min:s.

Group	Gender	*n*	Mean	SD	Minimum	25%	50%	75%	Max
World record	Men	8	02:03:52	00:01:19	02:01:39	02:03:16	02:03:48	02:04:33	02:06:05
	Women	3	02:25:05	00:08:25	02:19:46	02:20:14	02:20:43	02:27:45	02:34:48
Winner	Men	44	02:09:48	00:09:15	02:01:39	02:04:23	02:07:41	02:11:00	02:47:08
	Women	41	02:30:35	00:19:09	02:18:11	02:20:32	02:25:00	02:30:22	03:59:15
Top three	Men	132	02:11:06	00:09:26	02:01:39	02:06:14	02:08:34	02:12:00	02:50:02
	Women	123	02:34:59	00:25:51	02:18:11	02:23:38	02:26:49	02:32:00	04:39:24
Top 10	Men	440	02:14:22	00:10:30	02:01:39	02:09:09	02:11:58	02:14:00	02:58:55
	Women	404	02:40:55	00:29:44	02:18:11	02:27:25	02:32:36	02:38:20	05:40:10

Men were significantly faster than women in all performance groups (*p* < 0.05) and within the groups world record performances were significantly faster than top three and top 10 performances among both sexes (*p* > 0.05).

**Figure 1:**
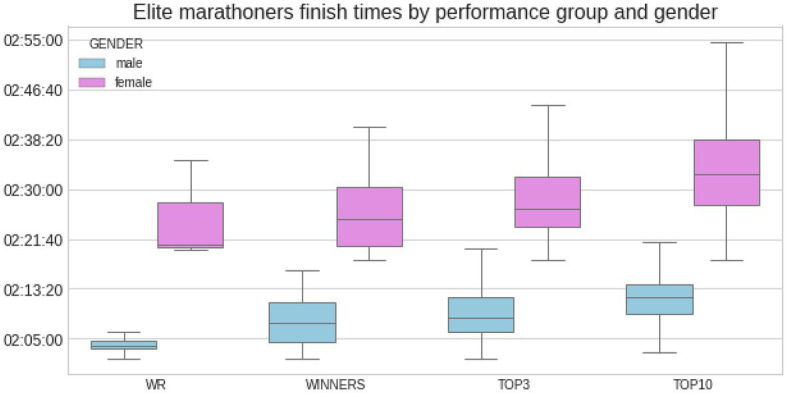
Marathon finishing times from world record (WR) performances and elite athletes (winner, top three, and top 10) by gender, with SD. Around 95% CI was used to create the boxplots. Statistical differences between male and female performances among all groups *p* < 0.05 and within sexes between WR and top three and top 10 performances.

**Figure 2:**
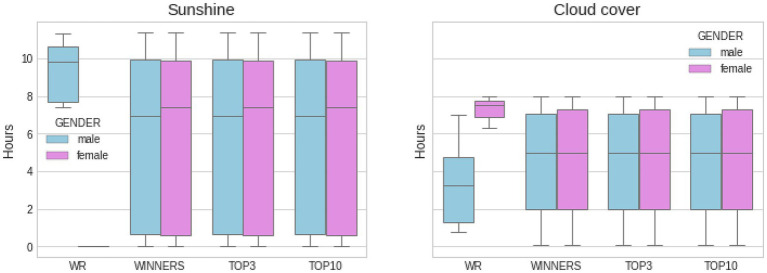
Comparative analysis of weather conditions (sunshine and cloud cover in hours) on WR marathon performances and elite marathon performances (winner, top three, and top 10) by gender, with SD. About 95% CI was used to create the boxplots. Statistical difference between WR men and women for both sunshine and cloud cover (*p* < 0.05).

**Figure 3:**
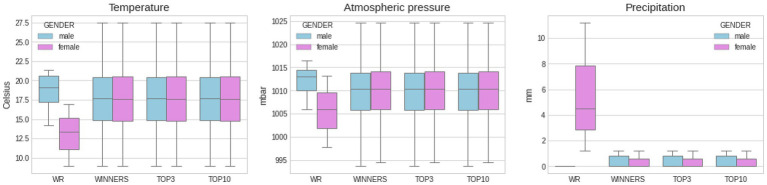
Comparative analysis of weather conditions [temperature (°Celsius), atmospheric pressure (mbar), and precipitation (mm)] on WR marathon performances and elite marathon performances (winner, top three, and top 10) by gender, with SD. Around 95% CI was used to create the boxplots. Statistical difference between WR female performance and precipitation to other performance groups (*p* < 0.05).

Figure 1 depicts and compares marathon finishing times from world record performances and elite athletes (i.e., winner, top three, and top 10) by gender. Figures 2, 3 show another set of boxplot charts illustrating a comparative analysis of the weather conditions [sunshine and cloud cover in hours and temperature (°Celsius), atmospheric pressure (mbar), and precipitation (mm)] on world record marathon performances and elite marathon performances (i.e., winner, top three, and top 10) by gender, with 95% CI. Temperatures were similar for the elite groups but lower for female world records and non-significant (*p* > 0.05) among all performance groups and sexes. Atmospheric pressure, precipitation, sunshine, and cloud cover were similar and not statistically different in elite marathon performances. Female world record-breaking performances occurred in bad weather (i.e., rain, cloud cover, and no sunshine; *p* < 0.05) compared to males.

**Figure 4:**
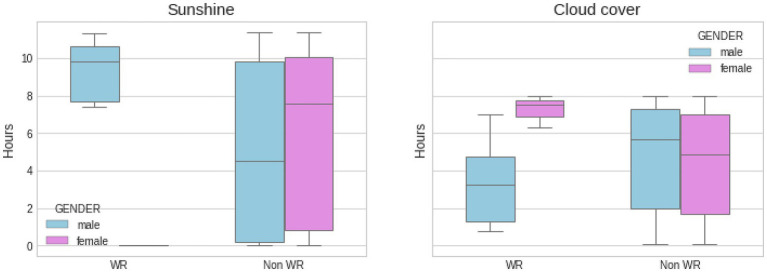
Comparative analysis of weather conditions (sunshine and cloud cover in hours) on WR marathon performances and non WR marathon performances by gender, with SD. About 95% CI was used to create the boxplots. Statistical differences between female WR and other groups for sunshine and cloud cover (*p* < 0.05).

**Figure 5:**
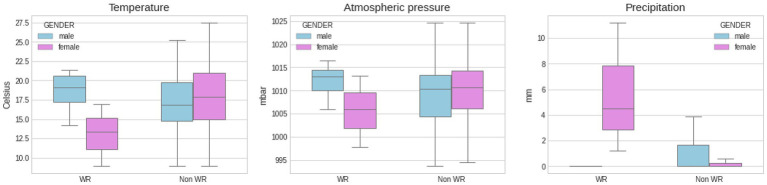
Comparative analysis of weather conditions [temperature (°Celsius), atmospheric pressure (mbar), and precipitation (mm)] on WR marathon performances and non WR marathon performances by gender, with SD. About 95% CI was used to create the boxplots. Statistical differences between female WR precipitation and all groups (*p* < 0.05).

Figures 4, 5 show another set of boxplots illustrating a comparative analysis of the weather conditions [sunshine and cloud cover in hours and temperature (°Celsius), atmospheric pressure (mbar), and precipitation (mm)] on world record marathon performances and non-world record marathon performances by gender, with 95% CI. The average temperatures for world record breaking events were 18.61 ± 2.59°C for men and 13.07 ± 4.01°C for women (*p* > 0.05), compared to 17.36 ± 4.33 and 17.93 ± 4.07°C for non-world record performances (*p* > 0.05), respectively for men and women, with no statistical difference between the groups. Male world records occurred on dry, high pressure days, while female world records were broken on days with lower barometric pressure (*p* > 0.05) and more precipitation (*p* < 0.05). The sunshine and cloud cover charts re-enforce further the idea that men performed better on sunny days, while women’s performance behaved rather the opposite way.

## Discussion

The aim of the present study was to investigate the influence of environmental conditions such as air temperature, sunshine duration, precipitation, barometric pressure, and cloud cover on world records and elite marathon race performances (i.e., winner, top three and top 10 finishers) at the “Berlin Marathon” between 1974 and 2019. In addition, we sought to describe the ideal weather conditions for world record performances and to compare performances between sexes. We hypothesized that temperatures of ~10–12°C would be the optimum temperature for both women and men to achieve a world record and elite performance in the “Berlin Marathon” and that fast marathon times were achieved in sunny weather, with little cloud cover and precipitation. The main findings were: (i) world record performances were set at a mean temperature of 18.61°C for men and 13.07°C for women, whereas elite performances were achieved at mean temperatures of 17.36 and 17.93°C for men and women (*p* > 0.05), respectively; (ii) hours of sunshine, cloud cover, precipitation, and atmospheric pressure were similar between male and female elite marathon performances (*p* > 0.05), and (iii) ideal weather conditions for world record performances differed between male and female athletes, where female athletes achieved world record performances with bad weather (i.e., rain and zero hours of sunshine and cloud cover), while males did so on sunny, mostly dry days, with higher atmospheric pressure and lower cloud cover.

### World Record Performances and Ambient Temperature

The “Berlin Marathon” is the fastest marathon racecourse in the world, with the current marathon world record for men obtained by Eliud Kipchoge in 2:01:39 h:min:s in 2018. In total, 11 world record performances were achieved at the “Berlin Marathon” since its inception in 1974. This represents 50% of all male and 10% of all female marathon world record performances worldwide since 1974. The reasons for this are said to be the cool climatic conditions, flat well-maintained roads, and carefully selected elite fields and pacemakers (Robinson, 2019); however, this has never been examined or confirmed in a scientific study. Indeed, the average temperature for world record at the “Berlin Marathon” were 18.61°C for men and 13.07°C for women, which can be classified as very warm thermal weather (Piotrowicz and Ciaranek, 2020). The temperatures are clearly higher than we expected, as previously ideal temperatures for elite performances were described in the range of between 8 and 12°C (Trapasso and Cooper, 1989; Martin and Buoncristiani, 1999; Ely et al., 2007b). Although the difference may be relatively small, a world record performance is unique and rare and this warmer temperature may be enough to make a difference in performance time. As a matter of fact, male world record athletes originate from Ethiopia and Kenya and they may be more accustomed to running in warmer, dryer weather with sunshine (Nikolaidis et al., 2017; Vitti et al., 2020). Additionally, these athletes have different body dimensions, with lower body mass and lower body mass index, that may be of advantage in higher temperatures (Mooses and Hackney, 2017).

### World Record Performances, Ambient Conditions, and Gender Differences

Ambient temperatures were sensibly different between male and female world record performances. There were also differences in other ambient conditions between world record performance and between sexes. While world record performances in men were held on mostly sunny and dry days, female world records were achieved in rather bad weather, with lower temperatures, rain and no sunshine, and this was statistically significant (*p* < 0.05).

These finding are novel and have not previously been reported in the literature. Cloud cover and/or solar radiation can be additional factors contributing to the likelihood of running a fast marathon race (Hughson, 1980; Morgan, 1980; Ely et al., 2007b), although no conclusive evidence exists (Bodishbaugh, 1988; Ely et al., 2007b). Our results provide the first evidence, that world record performances in female athletes at the “Berlin Marathon” are possible in “bad weather.” However, we also recognize that only three female athletes achieved a world record at the “Berlin Marathon,” so data interpretation is more challenging, and it may be speculative to draw firm conclusion from this. The female world records were set in 1977, 1999, and 2001. Especially in the Seventies of the last century, a female marathon world record could be achieved more easily than nowadays. Therefore, also rainy conditions may have not such a strong impact on the performance than in the male race of the last 2 decades.

However, these gender differences in respect to ambient conditions are interesting to note and represent a unique sample of performances. Larger sample sizes are needed to assess sex differences and environmental conditions and their impact on race performance. However, we also need to recognize that examining one marathon event is preferable to multiple marathon events at different race locations, as non-weather factors such as marathon racecourse can influence performance, hence this will always be a limitation to sample size.

Since the most recent male world records were set by Kenyan marathoners, the aspect of doping must be considered. In 2012, a German television report showed drug use by top Kenyan runners, and under international pressure, Kenyan antidoping task force reported a spike in doping cases in Kenyan runners at that time. It has been reported that 15 doped runners were marathoners, and most were sub-elite runners trying to become elite marathoners (Eichner, 2015).

### Elite Marathon Performances and Ambient Conditions

For elite performances, the ideal temperatures were of 17.36 ± 4.33°C for men and 17.93 ± 4.07°C for women. The average temperature range for elite performances at the “Berlin Marathon” was very warm thermal weather (Piotrowicz and Ciaranek, 2020), with no statistical differences between the sexes. These ambient temperatures are higher than we expected, and higher than the ones previously reported in the literature (between 8 and 12°C; Trapasso and Cooper, 1989; Ely et al., 2007b). Again, this may be related to the number of elite athletes originating from the African continent (Vitti et al., 2020), with the advantages of running in warmer weather and their described physiological advantages (Mooses and Hackney, 2017; Nikolaidis et al., 2017), or it may simply be an intrinsic advantage of the ambient conditions at the “Berlin Marathon.” However, it has also been shown, that elite women may benefit more from increasing temperatures for a fast marathon race time than men (Vihma, 2010). Other ambient conditions such as hours of sunshine, cloud cover, precipitation, and atmospheric pressure were similar between the sexes in elite marathon performances in our data, with ideal conditions of little to no precipitation, moderate cloud cover, and sunshine.

### Limitations

The methods of weather measurements might have changed during the decades, which also may have an impact on the results. The wet-bulb globe temperature (WBGT) as a combined index of ambient temperature, relative humidity, and solar radiation, has on occasion been used to examine performances (Ely et al., 2007b); however, the WBGT cannot distinguish between important weather variables, e.g., cloud cover or sunshine and rain on performance (e.g., a hot, dry, overcast day can result in a WBGT that is very similar to a cool and humid day with plenty of sunshine). Therefore, we have not analyzed data using the WBGT as our aim was to examine the different ambient conditions on performance separately. Wind speed may have an influence on performance on some marathon race courses (Vihma, 2010; Miller-Rushing et al., 2012; Knechtle et al., 2019); however, we are unable to assess this for the “Berlin Marathon,” as we did not have data on wind speed, but this would be another interesting aspect to examine in future studies. Although the historic weather data are mostly quality controlled by the “Deutscher Wetterdienst,” the use of historic weather data has its limitations. We used average humidity and average barometric pressure, sunshine duration, and precipitation for the whole day, but temperature respecting start times of the races (i.e., morning start in recent years, afternoon start in the world record race in 1977). However, future studies examining especially world record performances should bear this in mind. Hourly changes of weather data are obtainable from weather report data bases to correlate better with race start and race finish of athletes of each performance level (race time). The low number of world record performances make it difficult to generalize our results, especially in respect to a female world record. However, we also need to recognize that world record performances are rare and unique and that the “Berlin Marathon” provides the greatest number of world record performances of any singular marathon racecourse, therefore making it an ideal competition to investigate factors affecting these unique performances. Examining ambient conditions of world record performances from different racecourses will provide a larger dataset and may provide further information about sex differences in ambient conditions for these performances. An additional, historic limitation is that the characteristics of the race course changed from the beginning to the actual version. In the first years (1974–1980), the race was held outside of Berlin as a commute in the “Grunewald” and moved then to the city of Berlin. In the beginning, the racecourse was held in and near the forest covered by trees. The start times of the race have also changed over time, with a staged start used nowadays. As usual in a large city marathon, several aid stations are offered during the course and electronic time recording for block starts is used. Therefore, the first female world record in 1977 was held under different conditions (i.e., race start in the afternoon and course covered by trees) compared to the other female world records after 1981.

## Conclusion

The “Berlin Marathon” is the fastest marathon racecourse in the world and has seen 11 world records since it was first held in 1974. The ideal environmental conditions for world record performances for men were temperatures of 18.61°C, sunny, mostly dry days, with higher atmospheric pressure and little cloud cover. In women, ideal conditions for world record performances were temperatures of 13.07°C, with low atmospheric pressure, but significantly more rain, and with no sunshine and with cloud cover. No significant gender differences were observed in elite (i.e., winner, top three and top 10 finishers) performances at the “Berlin Marathon,” and the best ambient conditions in those are little to no precipitation, moderate cloud cover, and sunshine. For any athlete intending to break the marathon world record in an official race, optimum environmental conditions are needed apart from a fast race course. Future studies might consider the analysis of specific biomarkers during a marathon run in the heat (Lim et al., 2009; Suzuki, 2019) and the use of hourly weather data respecting race start and race finish.

## Data Availability Statement

The raw data supporting the conclusions of this article will be made available by the authors, without undue reservation.

## Author Contributions

VS drafted the manuscript. DV performed the data processing and statistical analyses. EV collected the data. JA, TR, and BK helped in drafting the manuscript. All authors contributed to the article and approved the submitted version.

### Conflict of Interest

The authors have no conflicts of interest to report. The authors confirm that the research presented in this article met the ethical guidelines, including adherence to the legal requirements.

## Footnotes

^1^www.bmw-berlin-marathon.com/news-center/news/detail/eine-einzigartige-geschichte-30-jahre-real-berlin-marathon/

^2^www.runnersworld.de/news-fotos/die-elf-berliner-marathon-weltrekorde/

^3^www.bmw-berlin-marathon.com/impressionen/statistik-und-geschichte/ergebnisarchiv/

^4^https://colab.research.google.com/
